# A Randomized Controlled Trial of an Enhanced Version of a Cognitive–Behavioral Video Game Intervention Aimed at Promoting Active Aging: Assessments of Perceived Health and Healthy Lifestyle Habits at Pre- and Post-Intervention

**DOI:** 10.3390/jcm14196873

**Published:** 2025-09-28

**Authors:** Tania Cotardo, Patricia Otero, Eling D. de Bruin, Vanessa Blanco, Manuel Arrojo, Mario Páramo, María J. Ferraces, Ángela J. Torres, Fernando L. Vázquez

**Affiliations:** 1 Department of Clinical Psychology and Psychobiology, University of Santiago de Compostela, 15782 Santiago de Compostela, Spain; fernandolino.vazquez@usc.es; 2 Department of Psychology, University of A Coruña, 15071 A Coruña, Spain; patricia.otero.otero@udc.es; 3 Motor Control and Learning Group, Institute of Human Movement Sciences and Sport, Department of Health Sciences and Technology, ETH Zurich, 8093 Zürich, Switzerland; eling.debruin@hest.ethz.ch; 4 Division of Physiotherapy, Department of Neurobiology, Care Sciences and Society, Karolinska Institutet, 17177 Stockholm, Sweden; 5 Department of Health, OST—Eastern Swiss University of Applied Sciences, 9001 St. Gallen, Switzerland; 6 Department of Evolutionary and Educational Psychology, University of Santiago de Compostela, 15782 Santiago de Compostela, Spain; vanessa.blanco@usc.es; 7 University Hospital Complex of Santiago de Compostela, Galician Health Service (SERGAS), 15706 Santiago de Compostela, Spain; manuel.arrojo.romero@sergas.es (M.A.); mario.paramo.fernandez@sergas.es (M.P.); 8 Department of Psychiatry, Radiology, Public Health, Nursing and Medicine, University of Santiago de Compostela, 15782 Santiago de Compostela, Spain; angelajuana.torres@usc.es; 9 Department of Social Psychology, Basic Psychology and Methodology, University of Santiago de Compostela, 15782 Santiago de Compostela, Spain; mjose.ferraces@usc.es

**Keywords:** active aging, video game, cognitive–behavioral intervention, graphic adventure, smartphone app

## Abstract

**Background/Objective:** Video games offer an innovative tool for delivering active aging interventions. The objective of this study was to analyze the efficacy of an enhanced version of a cognitive–behavioral intervention to promote active aging, administered through a video game, on perceived health and healthy lifestyle habits. **Methods:** A total of 556 participants aged 45 or older (74.3% women, mean age of 60.8 [*SD* = 8.0]) were randomly assigned to a cognitive–behavioral intervention to promote active aging administered via an interactive, multimedia, online, and serious video game with a complementary smartphone app (CBI-V; *n* = 279) or to a control group receiving non-specific online information (CG; *n* = 277). Perceived health (General Health, Body Pain, Physical Functioning, Physical Role, Vitality, Social Functioning, Emotional Role, Mental Health, Physical and Mental Summary Indices); healthy habits, including sleep hygiene behaviors, physical activity, and eating habits; dropouts; adherence to the intervention (completed modules; total playtime; time devoted to cognitive training; number of cognitive task attempts and tasks completed between modules); and satisfaction with the intervention were assessed by independent, blind evaluators via an online platform before and after the intervention. **Results:** At the post-intervention, the CBI-V group obtained significantly better scores in General Health (*p* < 0.001), Mental Health (*p* = 0.015), sleep hygiene (*p* < 0.001), physical activity (*p* = 0.024), and eating habits (*p* = 0.001) than the CG. The effect sizes were small (*d* = −0.188 to 0.334). Clinically significant changes were higher in the CBI-V group than in the CG in General Health (*p* = 0.029), Physical Function (*p* = 0.028), Social Function (*p* = 0.011), Emotional Role (*p* = 0.008), Mental Health (*p* = 0.026), Physical Summary Index (*p* = 0.010), sleep hygiene (*p* = 0.020), and eating habits (*p* = 0.019). Participants reported a high level of satisfaction with the intervention, with a mean score of 25.4 (*SD* = 4.0) out of a maximum of 32 on the intervention satisfaction measure. **Conclusions:** These findings support the efficacy of a cognitive–behavioral video game intervention in enhancing perceived health and promoting healthy lifestyle habits among people aged 45 and above.

## 1. Introduction

The phenomenon of aging populations is becoming increasingly prominent. According to the European Statistical Office [1], in Europe, 49.2% of the population is aged 45 or older, and 21.2% is aged 65 or older; these figures are expected to increase up to 53.7% and 29.0%, respectively, by 2050, which represents a socioeconomic and healthcare challenge.

Biologically, aging results from the build-up of molecular and cellular damage across the lifespan, leading to a loss of physical and mental functions, a higher susceptibility to disease, and ultimately, death [2]. There is evidence that aging does not follow a linear course and that there are specific time points at which biochemical changes accelerate [3,4]. The first abrupt and extensive molecular and microbiome changes occur around the age of 44 [3]. Thus, although there is considerable variability in individuals’ health trajectories during aging, the first physical changes associated with this process typically begin to manifest in middle age (approximately from the age of 45 onwards) [3,4,5]. These shifts include cardiovascular diseases; changes in lipid, caffeine, and alcohol metabolism; and alterations in skin and muscle [3]. Furthermore, neurobiological studies show that even in healthy adults, brain volume begins to decline at this age (particularly in the frontal and temporal cortices), accompanied by gradual reductions in memory, executive function, and processing speed [4]. Additionally, a longitudinal study found that numeric abilities and perceptual speed also decline in midlife [6].

Considering that only approximately 25% of those age-related changes are genetically determined, while the remaining 75% are affected by gene–environment interactions and lifestyle throughout the life course [7], midlife represents a strategic period to build an appropriate lifestyle that supports healthier aging trajectories and delays or prevents functional decline.

Previous studies have highlighted the critical role that healthy habits (adequate sleep, regular physical activity, and balanced nutrition) play in healthy aging. Sleep contributes to maintaining cognitive functions, emotional stability, and physical health among older adults [8]. Poor sleep quality and short sleep duration has been associated with adverse outcomes such as an increased risk of osteoarthritis (odds ratio (OR) = 1.73; 95% CI 1.33, 2.25) [9], cancer (hazard ratio (HR) = 1.59; 95% CI 1.15, 2.19) [10], heart attack (OR = 1.59; 95% CI 1.19, 2.13), cardiovascular diseases (OR = 1.42; 95% CI 1.13, 1,78), [11] and cognitive decline (OR = 2.19; 95% CI 1.10, 4.39) [12]. Regular physical activity can delay many of the negative effects of aging, improving both the physical and mental health of middle-aged and older adults. Aerobic exercise helps maintain arterial elasticity, control cholesterol levels, and regulate blood pressure by an average of 3.4/2.4 mmHg [13], and it reduces the risk of cardiovascular diseases (HR = 0.87; 95% CI 0.77, 0.99) [14]. Resistance exercise helps maintain muscle mass, improves strength, and supports physical function, all of which are essential for preserving independence in old age [15]. Moreover, it was found that individuals with enhanced capacity to generate synaptic plasticity were more physically active compared with those with less synaptic plasticity (*z* = −2.165, *r* = 0.36, *p* = 0.030) [16]. Additionally, moderate physical activity can reduce the risk of depression (HR = 0.71; 95% CI 0.63, 0.79) and anxiety (HR = 0.80; 95% CI 0.71–0.90) [17]. Lastly, healthy eating habits provide the necessary nutrients to support bodily functions and prevent age-related diseases. Specific dietary patterns, such as the Mediterranean diet, have been linked to a reduced risk of cardiovascular diseases (relative risks = 0.69; 95% CI 0.57, 0.83) [18]. Furthermore, adequate intake of vitamins and minerals is crucial for bone health. For example, it was found that persons with serum levels of 25-hydroxyvitamin D lower than 37.5 nmol/L were more likely to be frail than those between 50 and 75 nmol/L (OR = 1.47; 95% CI 1.19, 1.82); while those with serum 25-hydroxyvitamin D levels of at least 60 nmol/L were at lower risk of fractures than those with less than 30 nmol/L (HR = 0.69, 95% CI 0.57, 0.84 for nonvertebral fracture, and HR = 0.63, 95% CI 0.46, 0.87 for hip fracture) [19].

Due to these reasons, the WHO has emphasized the importance of active aging programs aimed at promoting healthy lifestyles across the life course to maximize the number of healthy and independent older adults [20]. One effective way to make these programs accessible is through technology-assisted interventions [21] such as video games. They are attractive, fun, can be used at home [22], and can overcome barriers of face-to-face programs (such as transportation).

There is growing evidence supporting the benefits of video game interventions for community-dwelling adults aged 45 and older [23]. For example, Kahlbaugh et al. [24] examined the impact of playing Nintendo Wii games (Nintendo Co., Ltd., Kyoto, Japan) on well-being in a sample of 35 older adults (average age 82 years). Individuals were randomly allocated to either the Wii gaming group (*n* = 16) or the group that involved watching TV with a partner (*n* = 12) over a 10-week period. The study observed that participants in the Wii group reported lower levels of loneliness (mean difference = −1 point, *p* < 0.05) and higher positive mood (mean difference = 3.6 points, *p* = 0.01) compared with the television group. No significant differences were identified in life satisfaction or physical activity; however, lower loneliness was a predictor of higher positive mood, which, in turn, predicted engagement in physical activity. In a separate study, Karahan et al. [25] examined the impact of exergames (i.e., exercise video games) on balance, functional mobility, and quality of life in 100 adults aged 65 and older. Participants were randomly assigned to a program using the Xbox 360 Kinect™ device (Microsoft Corporation, Redmond, WA, USA) (*n* = 48) or to the control group, consisting of a home exercise program on balance (*n* = 42), both for 6 weeks. Balance improved in both groups, but more in the intervention group (mean differences = 5.1 points vs. 1.7 points; all *p* < 0.001). Scores in the timed “up and go” test significantly improved only in the intervention group (mean difference = 0.6 points, *p* < 0.001). Moreover, the intervention group showed significant improvements for physical and social functioning, physical role, general health perception, and the physical component index (all *p* < 0.05), but no significant between-group differences were found. Similarly, Maillot et al. [26] investigated whether exergame training could improve braking force in walking and balance in 16 participants aged 65 or older. They compared an intervention group that played the Nintendo Wii video game (*n* = 8) with an untreated control group (*n* = 8) over a period of 12 weeks. The intervention group demonstrated significantly greater improvements compared with the control group in braking force during the normal step condition (*p* = 0.04), as well as in functional fitness indicators of lower limb strength, motor agility, cardiovascular endurance, and the global mental dimension of the SF-36 (all *p* < 0.001). Additionally, Duque et al. [27] assessed the efficacy of balance training delivered through a virtual reality system in 60 individuals aged 65 or older who suffered a prior fall. Participants were randomly assigned to a virtual reality program (*n* = 30) or to a control group (*n* = 30). Both received the usual fall prevention care for 6 weeks, but participants of the intervention group received additional balance training. Significant improvements were found in the intervention group, compared with the control group, in balance (mean difference on limits of stability = 50 cm^2^), falls (mean difference = 0.9 points), and fear of falling (mean difference = 1.5 points) (all *p* < 0.01).

However, previous research has also identified results contrasting with those described above. Montero-Alía et al. [28] analyzed the effects of video game-based balance training on 508 participants aged 70 years or older. The intervention group (*n* = 274) received balance training through the use of a Nintendo Wii console and was compared with a usual-care control group (*n* = 356) after 3 months. No differences were found between groups in balance or incident falls.

Collectively, findings from previous research suggest that video game-based interventions can provide benefits in older adults, specifically in aspects such as mental health, physical functioning, and balance. However, the findings are not completely consistent, and the scope of the effects differs according to the type of intervention, the characteristics of the sample, and the outcome measures. Possible reasons for this inconsistent findings include variability in sample size (e.g., ranging from 16 [26] to 508 participants [28]), participants age (e.g., ≥65 years [25,26,27], ≥70 years [28]), and recruitment setting (community [24,26], clinical settings [25,27,28]), which suggest differences in participants’ baseline health status; the heterogeneity of the video game-based interventions used; the frequency and duration of the interventions (e.g., from 30 min twice a week for 6 weeks [27] to 1 h twice a week for 12 weeks [26]); the delivery format (individual [24,25,26,27] vs. group-based [28]); and the types of devices used (e.g., Wii [24,26,28], Xbox 360 Kinect™ [25], virtual reality [27]).

Additionally, it is crucial to acknowledge additional limitations of previous research. They included samples of only adults over 60 years old, potentially diminishing the preventive effects of a healthy lifestyle across the course of life. In addition, most studies have neither based their interventions on a theoretical model nor used a manualized treatment (i.e., a therapeutic approach where the intervention is delivered according to a pre-defined, structured protocol or manual). The majority used exergames (i.e., those aimed at improving physical fitness by combining physical exercise with gameplay), and those that employed serious video games (i.e., designed to promote behavioral changes for educational or health purposes) focused exclusively on cognitive training. None of these video game-based interventions used a graphic adventure (i.e., narrative-based genre game with a plotline) nor used an app companion to extend the experience into the participant’s real life.

Considering the limitations of previous research, our team developed an innovative video game as an alternative to existing interventions aimed at promoting active aging. Unlike previous approaches, this proposal was based on a graphic adventure designed to encourage lifelong health promotion starting at the age of 45, offering an interactive experience focused on comprehensive improvement in well-being and lifestyle habits to promote active aging. In addition, it was the first video game to develop a complementary smartphone app, synchronized with the video game, that sent real-time reminders to the participant about the completion of tasks between sessions, which may improve adherence and the transfer of the skills acquired in the game to real-life situations. The pilot study [29] and randomized controlled trial (RCT) [30] phases of the initial version demonstrated the feasibility of the intervention and its efficacy on several subscales of perceived health, memory strategies used, and sleep hygiene. Subsequently, our video game intervention was reviewed and refined. A randomized controlled pilot study of the enhanced version of the video game [31] was conducted, and its results support its feasibility and encourage the evaluation of its efficacy in a larger-scale RCT, which led to the design of the present RCT. The performance of this RCT has generated a large amount of information, and given the breadth of the data collected, it was decided to present the most relevant findings in a progressive, organized, and detailed manner. Understanding that healthy habits (sleep, physical activity, and a healthy diet) are key factors in the promotion of active aging, this study prioritized the analysis of these variables due to their relevance and their potential practical impact on the design of interventions and strategies to promote a healthy lifestyle.

The primary aim of this study was to analyze the efficacy of the enhanced cognitive–behavioral intervention to promote active aging administered via an interactive, multimedia, online, serious video game in the graphic adventure genre with a complementary smartphone app on perceived health and healthy habits (sleep hygiene, physical activity, and eating habits). The intervention was expected to produce significant improvements in perceived health and healthy habits among participants in the intervention group compared with those in the control group.

## 2. Materials and Methods

### 2.1. Design

A parallel two-arm RCT was conducted (Trial registration: ClinicalTrials.gov. NCT04982497, https://clinicaltrials.gov/study/NCT04982497#study-record-dates, date of registration: 23 July 2021). Participants were randomly assigned, with an equal probability (1:1), to either an enhanced cognitive–behavioral intervention via the interactive, multimedia, online, serious video game with a complementary app (CBI-V) or a control group that received online information about active aging (CG), both accessed from home. The sequence of randomization was automatically generated by the evaluation platform (blinding of randomization). The evaluators and data analysts were blinded to group allocation throughout the study. However, participants were aware of their assigned group due to the nature of the interventions. This study follows the Consolidated Standards of Reporting Trials (CONSORT) statement guidelines [32].

### 2.2. Participants

Participants were recruited through eight media reports about the research project broadcasted in the press, radio, and television, senior university programs, and 20 socio-community associations from September 2021 to May 2024. To participate in this study, individuals had to meet the following criteria: (a) be 45 years of age or older; (b) show normal cognitive functioning, as determined by a cutoff point of 24 on the Mini-Mental State Examination (MMSE) [33,34]. The exclusion criteria comprised the following: (a) having serious mental or medical disorders (e.g., severe depression, schizophrenia, bipolar disorder, dementia, dissociative disorders, substance dependence, or acute suicidal ideation); (b) recent psychological or psychopharmacological treatment (in the past two months before the study), or participation in another trial related to active aging; (c) do not have the appropriate devices to play the game (computer and smartphone with internet connection), cannot communicate in Spanish, or have problems (e.g., sensory, physical) that make it impossible to play the video game.

This study was conducted in compliance with the latest version of the Declaration of Helsinki and was approved by the Bioethics Committee of the University of Santiago de Compostela, Spain (Code USC-38/2021). Participation was voluntary, and participants were not offered any rewards or incentives. All participants signed the written informed consent. Participant confidentiality was strictly maintained throughout the study. All personal identifiers were replaced with unique anonymized codes. All data were entered into a database without participants’ personal identification. The personal and clinical data of the participants were saved separately, and the linkage between these codes and individual participants was accessible only to authorized research personnel. Additionally, online assessment responses were collected through a secure platform using encrypted connections and stored on encrypted servers protected against unauthorized access. Access to the study data was restricted to researchers through a password system.

### 2.3. Sample Size

According to a meta-analysis [23] on the efficacy of video game-based interventions for active aging aimed at middle-aged to older adults, this study was powered to detect an effect size of 0.26 between the experimental and control group, assuming a bilateral alpha of 0.05, a power of 80%, and a sample loss of 8%. Therefore, the required estimated sample size was 548 participants (approximately 274 per group).

An interim analysis was conducted after enrollment of 50% of the target sample size and reviewed by the independent Data Monitoring Committee, who recommended continuation of the trial. It would be stopped if significant safety concerns were identified. To reduce participants’ attrition, strategies recommended by Cummings et al. [35] were followed: excluding individuals likely to be lost (based on the eligibility criteria); treating them with kindness, respect, and consideration; conducting noninvasive, engaging, and useful assessments; and motivating participants to remain in the trial.

### 2.4. Instruments

Independent trained interviewers (blinded to group allocation and random allocation sequence) completed the hetero-administered instruments at the pre-intervention measurement (before group allocation), and participants completed the self-administered instruments online both at pre- and post-intervention.

Exclusion of psychiatric disorders was determined via a clinical diagnostic interview with the Mini International Neuropsychiatric Interview (MINI) [36,37], with a sensitivity between 17% and 92% and a specificity between 75% and 100%. Cognitive function was evaluated using the MMSE [33,34], which has a sensitivity of 89.8% and a specificity of 75.1%.

Sociodemographic variables were collected at preintervention using an ad hoc questionnaire designed for this study.

Perceived health was assessed at both pre- and post-intervention using the Short-Form Health Survey (SF-36) [38,39]. This 36-item, self-administered instrument includes eight subscales (General Health, Body Pain, Physical Functioning, Physical Role, Vitality, Social Functioning, Emotional Role, and Mental Health) and two summary indices (Physical and Mental), with internal consistency values ranging from 0.71 to 0.94.

At pre- and post-intervention, sleep hygiene behaviors were assessed using the Sleep Hygiene Index (SHI; by Mastin et al. [40]), a 13-item, self-administered instrument with an internal consistency of 0.66. Physical activity was evaluated with the Brief Physical Activity Assessment Tool for Primary Care Consultations (BPAAT) [41,42], a two-item, self-administered measure which has test–retest reliability of k = 0.70. Eating habits were examined with the Rapid Eating Assessment for Participants-Short Version (REAP-S) [43], a 16-item, self-administered instrument which showed good convergent validity with the Food Frequency Questionnaire (*r* from −0.384 to 0.506).

Dropouts were documented. To assess the adherence to the intervention in the CBI-V group, the online video game and the app automatically recorded the number of completed modules, the total time spent playing, the duration devoted to cognitive training tasks, the number of attempts to solve cognitive tasks, and the number of completed tasks between modules. In the CG, only the number of completed modules was recorded. Satisfaction with the intervention was assessed with the Client Satisfaction Questionnaire (CSQ-8) [44,45], an 8-item, self-administered questionnaire with an internal consistency of 0.80. Adverse events were defined as any unfavorable and unintended symptom occurrence during the trial. Harms were recorded only if spontaneously reported by participants or observed by study staff during assessments.

### 2.5. Interventions

First, a protocol was created in advance of the research [46], and the intervention was manualized. An initial version of the intervention was developed within the framework of the National Project GAMAPEA (EXP-00091195/ITC-20161137) as an innovative, serious video game-based intervention aimed at promoting active aging from age 45 onward. It was tested in a pilot study [29] with 25 participants, which found a low dropout rate (8%); high adherence, satisfaction, and engagement; and significant improvements in General Health (*p* = 0.006, *d* = 0.56, 95% CI −0.01, 1.13), Physical Functioning (*p* = 0.007, *d* = 0.38, 95% CI −0.18, 1.94), Social Functioning (*p* = 0.003, *d* = 0.55, 95% CI −0.01, 1.12), and Mental Health (*p* = 0.006, *d* = 0.59, 95% CI 0.03, 1.16). An RCT [30] with 98 participants (44 in the intervention group and 54 in the control group) was then conducted to test the efficacy of the intervention. It found significant improvements in the intervention group compared with the control group in General Health (*p* = 0.005, *d* = −0.60, 95% CI –1.01, −0.19), Bodily Pain (*p* = 0.016, *d* = −0.50, 95% CI –0.91, −0.09), Vitality (*p* = 0.002, *d* = −0.65, 95% CI –1.06, −0.24), Social Functioning (*p* = 0.010, *d* = −0.53, 95% CI –0.94, −0.13), Physical Health Index (*p* = 0.045, *d* = −0.42, 95% CI –0.82, −0.01), frequency of mnemonic strategy use (*p* = 0.035, *d* = −0.44, 95% CI –0.85, −0.03), and sleep hygiene (*p* = 0.004, *d* = 0.61, 95% CI 0.20, 1.02). These findings supported the potential of this video game as an innovative tool for promoting active aging.

Subsequently, the video game underwent a comprehensive review and improvement process as part of the GAMEPROAGING National Project (PID2019–105052RB-I00, AEI/10.13039/501100011033). The content of the intervention was improved, and various aspects of the game were refined to enhance its effectiveness, optimizing its mechanics, strategies, and overall user experience. A randomized controlled pilot study on the enhanced version of the cognitive–behavioral video game intervention [31], with 55 participants (29 and 26 in the intervention and control groups, respectively), found a low dropout rate (3.6%), high adherence (with no differences between groups), and high engagement and satisfaction with the intervention. There were significant improvements in the intervention group, compared with the control group, in General Health (*p* = 0.0386, *d* = 0.63, 95% CI 0.23, 1.02), Vitality (*p* = 0.0283, *d* = 0.75, 95% CI 0.34, 1.16), Social Functioning (*p* = 0.0130, *d* = 0.71, 95% CI 0.31, 1.12), and Physical Summary Index (*p* = 0.0370, *d* = 0.56, 95% CI 0.17, 0.95). These findings support the feasibility of the enhanced version of the video game and justify the evaluation of its efficacy in an RCT.

Thus, the intervention presented in this article is the enhanced version of that previously developed and tested [29,30], the feasibility of which was demonstrated in a prior randomized controlled pilot study [31].

#### 2.5.1. Cognitive–Behavioral Intervention via Interactive Multimedia Online Serious Video Game with Complementary Smartphone App (CBI-V)

The intervention group received a cognitive–behavioral intervention to promote active aging, delivered through an interactive, multimedia, online, and serious video game (named GAMAPEA) with a complementary smartphone app. The intervention comprised eight modules, each lasting approximately 70 min to play, and was administered one module per week. During the intervals, participants were assigned tasks to complete with the aim of generalizing the skills taught in the video game to real-life situations. The intervention addressed the following three key components: depression prevention, promotion of healthy lifestyle habits, and cognitive training.

The prevention of depression component was based on the multifactorial etiopathogenic model of Lewinsohn et al. [47], which has demonstrated its efficacy in previous RCTs, both short- and long-term, across various administration formats: face-to-face [48,49], telephone conference calls [50,51], and smartphone app [52]. This component included techniques such as mood monitoring, methods for controlling psychophysiological arousal, behavioral activation, self-reinforcement, and strategies to foster positive thoughts. The healthy lifestyle habits component was grounded in the learning theory and social cognitive theory model [53,54] and incorporated techniques such as psychoeducation, self-observation, self-reinforcement, modeling, goal setting, developing a change plan, identifying barriers, and problem solving, as well as feedback. The cognitive training component was grounded in cognitive reserve [55] and neuroplasticity [56] to mitigate memory deficits associated with aging [57], such as reduced processing capacity, difficulties in semantic encoding, and challenges in information retrieval. The games were designed to stimulate attention, memory, language, and executive functions (e.g., planning, attention control, decision-making, flexibility) and included strategies to support data encoding, storage, and retrieval of information. Encoding strategies included acronyms, grouping, visual imagery, narrative linking. Storage strategies included repetition and focused attention on details. Retrieval strategies included external aids such as agendas, notes, and visible placement of objects.

As detailed in Table 1, the first module introduced psychoeducation on depressive symptoms and the importance of active coping, including mood self-monitoring, and training in diaphragmatic breathing for arousal regulation. Modules two and three focused on behavioral activation, incorporating strategies such as structuring, scheduling, and monitoring pleasant activities. Modules three through five introduced psychoeducation on healthy lifestyle habits (sleep, physical activity, and nutrition), along with strategies for developing them (e.g., sleep hygiene, physical activity records, and stimulus control techniques). Modules six and seven addressed cognitive restructuring, training participants to identify negative thoughts and replace them with more rational and positive ones using techniques such as direct approach or survey. Module eight targeted self-esteem enhancement and relapse prevention, reviewing previous content and consolidating strategies to maintain gains. Cognitive and social skills training were integrated throughout all modules.

The type of video game developed was a graphic adventure based on the “French Way” of the Way of Saint James (from Roncesvalles to Santiago de Compostela). The main character (Jacobo) was relatable to the participants. The game immerses the player in a narrative, including interactions with other characters, environmental elements, and problem-solving tasks, aiming the player to acquire psychological tools and train cognitive abilities.

The complementary smartphone app was synchronized with the video game, allowing users to review what they learned, the stages of the Way of Saint James that they completed, record tasks between modules, receive reminders (notifications), and track their progress.

#### 2.5.2. Control Group (CG)

A control group was used to account for contextual and non-specific factors such as participating in a study, the effect of time, and using a technological device. It was aligned with the CBI-V group regarding delivery format (online), number of modules (eight), delivery (a pace of one per week), duration of modules (around 70 min), and the psychoeducational content. The control group participants received general information to read about active aging without specific change strategies. As detailed in Table 1, the first module covered the topic of “active aging”. Module two focused on depression. Modules three to five explored healthy lifestyle habits (sleep, physical activity, balanced nutrition). Module six discussed negative thoughts. Module seven addressed cognitive processes, and module eight examined social relationships.

### 2.6. Data Analysis

Statistical analyses were performed using SPSS Statistics (version 27.0) and R. All analyses adhered to the intention-to-treat principle, and missing data were handled using the multiple imputation procedure according to Rubin’s method [58] with the EBM algorithm of the Amelia II program, resulting in 10 imputations. An imputation model was determined for each variable, ensuring that 10% of intervals that exclude the y = x line were not exceeded.

Differences between the intervention and control groups in perceived health (each subscale and summary index) and healthy lifestyle habits (sleep hygiene, physical activity, eating habits) were analyzed using linear mixed models. Mixed models, including the time factor, group, and time × group interaction, were analyzed. Bonferroni correction was used in the post hoc contrasts. Effect sizes were estimated using Cohen’s *d*, considering *d* ≥ 0.20 as small, *d* ≥ 0.50 as medium, and *d* ≥ 0.80 as large [59]. The lme4 package in R was used to fit mixed models, and the emmeans package was used to estimate means and contrasts.

The clinical significance of the intervention effects was determined following the formula by Jacobson et al. [60,61] to find a cut-off point (c) at which the subject’s post-intervention score falls within the distribution of the functional population: c = (*SD*_0_ *M*_1_ + *SD*_1_ *M*_0_)/*SD*_0_ + *SD*_1_, where *SD*_0_ = *SD*_1_ = pre-intervention (experimental or control group) or general population standard deviation; *M*_0_ = functional general population mean; *M*_1_ = pre-intervention mean (experimental and control group). Differences between groups in the percentages of clinically significant change were analyzed using the Chi-square statistical test.

Frequency analysis and descriptive statistics were conducted to describe dropouts, the number of completed modules, total time spent playing, time devoted to cognitive training tasks, the number of attempts to solve cognitive training tasks, completed tasks between modules, and satisfaction with the intervention. Differences between groups were analyzed using Student’s *t*-test for independent samples for continuous variables and Chi-square for categorical variables.

## 3. Results

### 3.1. Participant Flow

**Figure 1:**
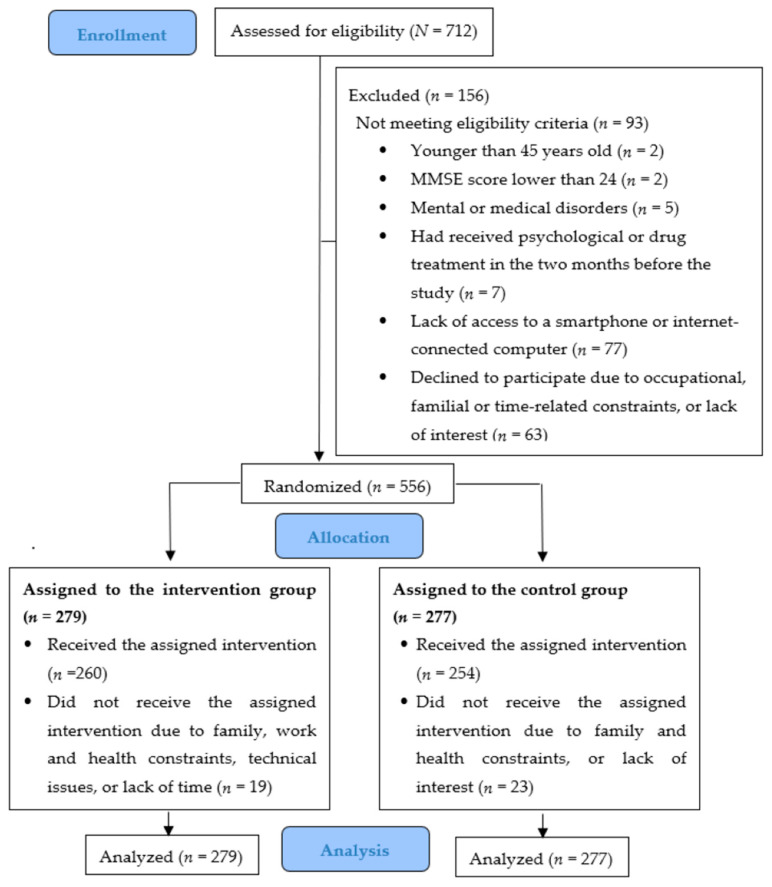
CONSORT flow diagram of participants of the study.

Figure 1 shows that of the 712 subjects assessed for eligibility, 93 (13.1%) did not meet the eligibility criteria, and 63 (8.9%) declined to participate. The final sample included a total of 556 participants randomized to the two groups: 279 to the CBI-V and 277 to the CG. All of them were analyzed according to the intention-to-treat principle.

### 3.2. Sociodemographic Characteristics of the Sample

In Table 2, the sociodemographic characteristics of the sample are shown. Of the participants, 74.3% were women, and the mean age was 60.8 years (*SD* = 8.0); 64.6% had a partner; 58.5% had a university-level education; 55.2% were homemakers, unemployed, or retired; and 54.5% had a monthly family income ≥EUR 2000. No statistically significant differences between the CBI-V and CG were observed in the sociodemographic variables.

### 3.3. Effects of the Intervention

#### 3.3.1. Perceived Health

Statistically significant effects were found for the group factor in Mental Health, (*F*(1, 554) = 4.49, *p* = 0.035); for the time factor in General Health (*F*(1, 554) = 4.28, *p* = 0.039) and Mental Health (*F*(1, 554) = 4.80, *p* = 0.029); and for time × group interaction in General Health (*F*(1, 554) = 14.59, *p* < 0.001), Physical Functioning (*F*(1, 554) = 6.66, *p* = 0.010), and Physical Summary Index (*F*(1, 554) = 10.31, *p* = 0.001).

Regarding intragroup changes (see Table 3), in the CBI-V group, there were significant increases from the pre- to post-intervention in General Health (*t*(742) = −3.836, *p* < 0.001, *d* = −0.195, 95% CI −0.295, −0.095) and Mental Health (*t*(813) = −2.469, *p* = 0.014, *d* = −0.138, 95% CI −0.247, −0.028); whereas in the CG, there was a significant decrease in Physical Functioning (*t*(728) = 2.115, *p* = 0.035, *d* = 0.110, 95% CI 0.008, 0.212) and Physical Summary Index (*t*(757) = 2.478, *p* = 0.013, *d* = 0.132, 95% CI 0.027, 0.236). No significant changes were observed in the rest of the subscales.

Regarding between-group changes, there were no significant differences in any subscale of SF-36 at the pre-intervention time point between the CBI-V group and the CG. At post-intervention (see Table 4), the CBI-V group attained significantly higher scores compared with the CG in General Health (*t*(742) = −2.760, *p* < 0.001, *d* = −0.248, 95% CI −0.424, −0.072) and Mental Health (*t*(813) = −2.45, *p* = 0.015, *d* = −0.218, 95% CI −0.392, −0.043). No statistically significant differences were obtained across the remaining subscales.

#### 3.3.2. Healthy Habits

Statistically significant effects were found for the time factor in sleep hygiene (*F*(1, 554) = 27.40, *p* < 0.001), physical activity (*F*(1, 554) = 17.86, *p* < 0.001), and eating habits (*F*(1, 554) = 27.57, *p* < 0.001) and for the time × group interaction in sleep hygiene (*F*(1, 554) = 12.40, *p* < 0.001), physical activity (*F*(1, 554) = 14.43, *p* < 0.001), and eating habits (*F*(1, 554) = 17.73, *p* < 0.001).

Regarding intragroup changes (see Table 3), in the CBI-V group there were significant improvements between the pre- and post-intervention in sleep hygiene (*t*(815) = 5.959, *p* < 0.001, *d* = 0.319, 95% CI 0.214, 0.424), physical activity (*t*(875) = −5.423, *p* < 0.001, *d* = −0.322, 95% CI −0.439, −0.206), and eating habits (*t*(948) = −6.122, *p* < 0.001, *d* = −0.419, 95% CI −0.553, −0.285), whereas in the CG, no statistically significant differences were observed in any of the measured lifestyle habits.

Regarding between-group changes, no significant differences were found at the pre-intervention. At post-intervention (see Table 4), the CBI-V group achieved significantly better scores than the CG in sleep hygiene (*t*(815) = 3.779, *p* < 0.001, *d* = 0.334, 95% CI 0.160, 0.507), physical activity (*t*(875) = −2.261, *p* = 0.024, *d* = −0.188, 95% CI −0.351, −0.025), and eating habits (*t*(948) = −3.205, *p* = 0.001, *d* = −0.291, 95% CI −0.469, −0.113).

### 3.4. Clinically Significant Change

#### 3.4.1. Perceived Health

A significantly higher percentage of participants in the CBI-V group compared with the CG experienced a clinically significant change in General Health (68.1% vs. 59.2%, respectively; χ^2^ = 4.75, *p* = 0.029), Physical Functioning (82.1% vs. 74.4%; χ^2^ = 4.85, *p* = 0.028), Social Functioning (55.9% vs. 45.1%; χ^2^ = 6.47, *p* = 0.011), Emotional Role (71.3% vs. 60.6%; χ^2^ = 7.06, *p* = 0.008), Mental Health (76.0% vs. 67.5%; χ^2^ = 4.93, *p* = 0.026), and Physical Summary Index (78.9% vs. 69.3%; χ^2^ = 6.59, *p* = 0.010) but not in the other subscales (Table 5).

#### 3.4.2. Healthy Habits

The percentage of participants who reached a clinically significant change was higher for the CBI-V group than the CG in sleep hygiene (99.3% vs. 96.4%, respectively; χ^2^ = 5.39, *p* = 0.020) and eating habits (67.0% vs. 57.4%; χ^2^ = 5.48, *p* = 0.019), but there was no difference in physical activity (Table 5).

### 3.5. Dropouts, Adherence, and Satisfaction with the Intervention

#### 3.5.1. Dropouts

Only 42 (7.6%) participants dropped out of the study — 19 (6.8%) from the CBI-V group and 23 (8.3%) from the CG—, without significant differences between groups (χ^2^ = 0.44, *p* = 0.505). Participants reported the following reasons for withdrawing from the study: family, work, health problems, technical difficulties, time constraints, or limited interest.

#### 3.5.2. Adherence

The average number of completed modules was 7.9 (*SD* = 0.4) out of 8 in the CBI-V group and 7.8 (*SD* = 0.4) in the CG, with no significant differences, *t*(506.47) = −1.719, *p* = 0.086. In the CBI-V, participants spent, on average, 73.8 min (*SD* = 23.5) on each module and 587.0 min (i.e., 9 h 47 min; *SD* = 93.6) on the total game. A mean of 21.6 min (*SD* = 15.2) per module and 172.6 min (i.e., 2 h, 58 min; *SD* = 69.2) in total were devoted to cognitive training tasks. Specifically, a mean of 28.6 min (*SD* = 14.5) was dedicated to attention, 58.9 min (*SD* = 29.0) to memory, 13.5 min (*SD* = 7.2) to language, and 69.6 min (*SD* = 38.4) to executive functions. On average, participants made 3.2 (*SD* = 3.7) attempts to solve each cognitive task per module, totaling 19.0 times (*SD* = 22.2) across the entire video game. Lastly, an average of 201.3 (*SD* = 28.9) tasks out of 249 throughout the intervention were completed between modules.

#### 3.5.3. Satisfaction

The average satisfaction score with the intervention was 25.4 (*SD* = 4.0) out of 32. In the CSQ-8 items, it was found that 90.6% of participants rated the service’s quality as good or excellent, and 95.7% considered that, in general or without a doubt, it was the type of service they wanted. A total of 61.0% stated that the program met most or almost all of their needs, and 91.0% stated that they would recommend it to a friend. A total of 93.2% of participants were satisfied or very satisfied with the amount of help received, and 91.8% claimed that the services received did help them to better manage their problems. Finally, 93.2% were satisfied or very satisfied with the service received during the intervention, and 85.3% believed they would repeat it or would repeat it without hesitation.

## 4. Discussion

### 4.1. Principal Findings

The present study reports the findings on perceived health and healthy habits of the enhanced version of the cognitive–behavioral intervention to promote active aging administered through an interactive, multimedia, online, and serious video game in a graphic adventure genre with a complementary smartphone app compared with a control group. The results support the efficacy of the enhanced intervention on participants’ perceived health and healthy lifestyle habits.

Specifically, in the CBI-V group, a significant improvement between the pre- and post-intervention was observed in General Health and Mental Health, while in the CG, a significant decline was found in Physical Functioning and Physical Summary Index. At post-intervention, compared with the CG, the CBI-V group had significantly higher scores in General Health and Mental Health, with small effect sizes (*d* = −0.248 and *d* = −0.218, respectively), in line with the effect sizes obtained in universal prevention serious video games for active aging [23]. Our findings surpass those of Kahlbaugh et al. [24], who reported nonsignificant improvements after a video game-based intervention in the overall perceived health assessed with the SF-36. Another study [25] detected improvements in various SF-36 subscales in within-group analysis of the intervention group, but did not detect significant changes in between-group differences with the control group. A study by Maillot et al. [26] only found differences in the between-group analysis in the global mental subscale. One possible explanation for our positive findings in different SF-36 subscales was the multi-component nature of the intervention (including depression prevention, healthy lifestyle promotion, and cognitive training components), which allowed improvements in both physical and mental health, whereas the other studies employed exergames focused solely on physical activity. Other possible explanations include the larger sample size in our study (*n* = 556), which provided greater statistical power compared with previous studies (sample sizes ranging from 16 [26] to 100 [25] participants), and the inclusion of younger participants (aged 45 and above) rather than exclusively older adults (≥65 years) as in prior research. Moreover, unlike previous studies, our intervention incorporated tasks between modules, which may have promoted greater dedication, skill refinement, and generalization of the learned abilities to participants’ daily lives beyond the game, thereby enhancing their perceived health-related quality of life.

Additionally, the CBI-V group exhibited a significant improvement in sleep hygiene, physical activity, and eating habits from pre-to post-intervention, whereas no significant changes were found in the CG. At post-intervention, the CBI-V group obtained significantly better scores than the CG in sleep hygiene, physical activity, and eating habits, with small effect sizes (from *d* = −0.188 to *d* = 0.334). These outcomes are more favorable than those found by Duque et al. [27], who did not report significant between-group differences in attending an exercise program for balance training and taking nutritional supplements. A possible explanation of our findings is that the gamification of the intervention enhanced motivation and engagement with the treatment through the fantasy immersion, interactivity, and modeling, which increase changes in health-related behaviors [62].

The percentage of participants exhibiting clinically significant changes was significantly higher in the CBI-V group than in the CG in General Health, Physical Functioning, Social Functioning, Emotional Role, Mental Health, Physical Summary Index, sleep hygiene, and eating habits. Although previous studies have not assessed clinical significance and we cannot compare studies, these findings suggest that our results have important clinical implications, since they reflect the extent to which the intervention impacts participants’ daily life. Based on our data, we calculated the number needed to treat (NNT) from the absolute risk reduction (ARR) in participants who did not achieve a clinically significant change [63], finding values ranging from 9 in Social Functioning and Emotional Role to 34 in sleep hygiene, with most variables showing NNTs ≤ 12, meaning that we have to provide the video game to 12 individuals to reach one additional positive outcome. In the context of a health promotion intervention —rather than clinical treatment— these results are encouraging. While clinical interventions typically aim for low NNTs because of the presence of diagnosable conditions, health promotion programs target the general or at-risk population, where the expected changes are often smaller in magnitude. Higher NNTs may still be acceptable in public health when interventions are low-cost, low-risk, and scalable, especially if they have the potential to reach large populations [64]. Thus, the current findings support the viability of the video game as a public health intervention and suggest that its effectiveness could be enhanced through further iterative development incorporating components of training with ecological validity [65].

Only 7.6% of the participants discontinued the study, a rate lower than those reported in other active aging interventions administered through video games, which reached up to 36.0% [28]. Moreover, participants’ adherence and satisfaction with the intervention were high, with a mean of 7.9 out of 8 completed modules and an average of 201.3 out of 249 completed tasks between modules. These results may be explained by diverse factors. First, the format of the intervention facilitated accessibility due to the flexibility to receive the intervention at the time and place most convenient to the participants. Second, the complementary smartphone app synchronized with the video game and sent reminders to access the next module and to complete between-module tasks, serving as a stimulus induction to encourage adherence. Additionally, the research team made contact to encourage continuation of the assigned program when a participant did not make progress. Third, the fact that the intervention was gamified may increase the intervention’s enjoyment and appeal for the participants, thereby enhancing their motivation and overall satisfaction. Fourth, the level of difficulty of the challenges that the players faced during the game was gradually increased, and their progress was reinforced. The setting (the Way of Saint James) allowed identification with meaningful geographical, cultural, and historical elements. Lastly, the graphic adventure video game, with a storyline and a slower pace, is usually preferred by older adults [66] compared with faster-paced games, likely due to their slower processing speeds [67].

### 4.2. Implications

This study has relevant implications for research, clinical practice, and social policies. It demonstrates the efficacy of the enhanced version of the cognitive–behavioral program applied through a video game to promote active aging on perceived health and healthy habits. Its innovative format can increase the attractiveness and facilitate the accessibility of the intervention, given that the majority of people of this age group have the technology needed to participate: a total of 96% of people 50 to 64 years old and 75% of those 65 years and older are internet users [68], and more than 98% of those 45 to 54 years old, 93% of those 55 to 64 years, and 77% of those 65 years and older have a smartphone [69]. Given the continuing growth of the aging population, public health policies are needed to establish effective interventions to promote active aging, such as the one presented in this study.

### 4.3. Limitations

However, this study also has some limitations. In the recruitment process, the requirement to have access to the internet or a smartphone excluded groups that are less familiar with technology or disadvantaged groups. Although most middle-aged and elderly people have access to the internet or a smartphone, this could introduce selection bias. In addition, the middle-aged subsample (45 to 65 years old) could have partially influenced the results, such as an overall better perceived health. However, it should be noted that this equally affected both groups, so the efficacy of the intervention remains proven. The absence of long-term follow-up prevents determining whether the effects of the intervention persist over time. Future research with extended follow-up periods is warranted to assess the durability of these findings. Moreover, this study was conducted in Spain, and the cultural and historical references of the Way of St. James may not be transferable elsewhere, so the results may not be generalizable to other countries. Finally, the study did not identify which subgroups of participants may derive the greatest benefit from the intervention. Differences in age, baseline health status, or psychosocial factors may influence outcomes, and future research should investigate these potential moderators. Additionally, while the overall efficacy of the intervention has been demonstrated, the specific mechanisms through which each component contributed to the observed effects remain unclear. Future studies should conduct mediation analyses to disentangle the relative contributions of individual components (depression prevention component, healthy lifestyle habits component, or cognitive training component).

## 5. Conclusions

These findings demonstrate the efficacy of the enhanced cognitive–behavioral intervention administered through an interactive, multimedia, online, and serious video game, complemented by a smartphone app, in promoting active aging by improving perceived health and healthy lifestyle habits. The results support the further development of this approach and encourage additional research into the efficacy and long-term impact of the video game.

## Figures and Tables

**Table 1:** Main contents of the cognitive–behavioral video game intervention with a complementary smartphone app and the control group.

Module	Intervention Group			Control Group
**1**	**From Roncesvalles to Pamplona.** The main character (Jacobo) is presented. He is depressed and starts the French Way of the Santiago de Compostela pilgrimage. He receives a (magical) book that guides him through the stages, explaining the interconnections between behavior, thoughts, and emotions. Jacobo encounters characters who provide him with lessons and guidance, such as monitoring his mood, relaxation techniques, and cognitive training tasks (puzzles).	**Contents:** Emotional regulation Mood self-monitoring Learning to relax **Techniques/strategies:** - Psychoeducation. Information about depressive symptoms and the importance of active coping - Mood self-monitoring and self-recording - Relaxation training: diaphragmatic breathing (training through modeling) - Mnemonic strategies (association, repetition), visual and verbal memory tasks - Social skills, assertive communication - Positive reinforcement when completing microgames	**Tasks between modules (app):** Daily self-monitoring of mood Relaxation through diaphragmatic breathing	**Aging** **Contents:** Population aging The aging process Consequences of aging Active aging
**2**	**From Pamplona to Estella**. Jacobo learns about the legend of the Reniega Fountain. Suddenly, he is attacked by Belfegor (one of Lucifer’s henchmen), who steals the book. To get the book back, Jacobo is given instructions to find nine sets of ‘‘emotional vitamins’’ hidden within scallop shells that symbolize the Way. For this purpose, he must complete cognitive training tasks. Finally, he gets the book back. Another pilgrim teaches him self-reinforcement and how to apply it.	**Contents:** Behavioral activation **Techniques/strategies:** - Feedback on task completion - Long-term memory: review of the previous module - Pinpoint pleasant activities - Self-reinforcement - Mnemonic strategies (repetition, creating a story, grouping, association, taking notes), verbal fluency tasks, visual tracking, cancelation, ideomotor praxis, short-term memory, verbal and visuospatial work - Social skills, assertive communication - Positive reinforcement when completing microgames	**Tasks between modules (app):** The previous ones plus the following: Elaborating on a list of pleasant activities	**Depression** **Contents:** What is depression? Prevalence of depression Evolution of depression Consequences of depression Conclusions
**3**	**From Estella to Nájera.** Jacobo experiences difficulties with sleep, and different characters provide him with strategies to improve his sleep hygiene. They also teach him how to schedule pleasant activities and establish behavioral contracts with himself. Through these efforts, he declares war on Dysnergy. To defeat her, he must find the Tau cross by solving cognitive training tasks. In the end, Jacobo battles and prevails over Dysnergy.	**Contents:** Learning healthy sleep habits Increasing pleasant activities **Techniques/strategies:** - Feedback on task completion - Long-term memory: review of the previous module - Scheduling pleasant activities - Sleep: Psychoeducation. Sleep hygiene, stimulus control - Mnemonic strategies (chaining), verbal reasoning, working memory, executive functions (planning), spatial orientation - Social skills, assertive communication - Positive reinforcement when completing microgames	**Tasks between modules (app):** The previous ones plus the following: Implementing scheduled pleasant activities	**Sleep** **Contents:** Sleep phases Changes in sleep related to aging Main sleep problems Prevalence of sleep problems Treatment of sleep problems Conclusions
**4**	**From Nájera to Burgos.** Jacobo’s mood is improving. Jacobo and Guillén (a fellow pilgrim) are ambushed by Dysnergy and Moribilius, who steal the book. With the support of Templar monks, they fight back and receive recommendations on physical activity for maintaining a healthy physical condition. To progress, cognitive training tasks (puzzles) must be solved. Later, they fight against Dysnergy, defeat her, and recover the book. However, Morbilius manages to escape.	**Contents:** Building a personal physical activity plan **Techniques/strategies:** - Feedback on task completion - Long-term memory: review of the previous module - Scheduling pleasant activities - Physical activity: Psychoeducation. Self-monitoring, contingency management, intrinsic motivation, self-control strategies (setting intermediate and final goals), action planning (what, when, where with whom, and how) - Verbal working memory, executive function (planning), ideational praxis - Social skills, assertive communication - Positive reinforcement when completing microgames	**Tasks between modules (app):** The previous ones plus the following: Implementing the personal plan to accomplish the weekly physical activity goal Self-reinforcement	**Physical Exercise** **Contents:** Sedentary lifestyle Physical activity Physical activity in middle to old age Relevance of physical activity for health Conclusions
**5**	**From Burgos to Sahagún.** After solving cognitive tasks (puzzles), the book provides key information on healthy eating habits. Jacobo and Guillén meet Culinuris. Morbilius appears, kidnaps Culinuris’ niece, and steals the book. Culinuris states that they must find three colored feathers to defeat Morbilius. Jacobo and Guillén obtain them after completing memory exercises focused on healthy eating. They then confront Morbilius, defeat him, rescue the niece, and get the book back. Guillén decides to settle down, while Jacobo continues his pilgrimage.	**Contents:** Improving dietary habits **Techniques/strategies:** - Feedback on task completion - Long-term memory: review of the previous module - Healthy eating: Psychoeducation. Intrinsic motivation, essential information, improvement plan, meal planning - Mnemonic strategies (association, categorization, repetition, acronym), visual and working memory, ideomotor praxis, executive function (planning) - Social skills, assertive communication - Positive reinforcement when completing microgames	**Tasks between modules (app):** The previous ones plus the following: Self-recording of daily meals	**Diet** **Contents:** Diet Healthy eating Healthy eating in middle to old age Conclusions
**6**	**From Sahagún to Rabanal.** The book explains the role of thoughts, their impact on mood, and how to detect dysfunctional thoughts. Jacobo encounters the architect Antón Gaudín, who supports him in addressing cognitive distortions. They join forces to battle the gargoyles of the Episcopal Palace of Astorga. Afterwards, Jacobo practices these cognitive restructuring skills with fellow pilgrims at the hostel.	**Contents:** Relationship between thoughts and mood Cognitive distortions (polarized thinking, catastrophizing, personalization) **Techniques/strategies:** - Feedback on task completion - Long-term memory: review of the previous module - Psychoeducation, Self-observation and self-recording of negative thoughts - Mnemonic strategies (rhyme), attention and concentration, language, verbal and visual memory, ideomotor praxis - Social skills, assertive communication - Positive reinforcement when completing microgames	**Tasks between modules (app):** The previous ones plus the following: Daily tracking and personalized feedback to improve eating habits Detection of negative thoughts	**Thoughts** **Contents:** The structure of thought Relationship between thought and mood Types of thoughts Thoughts in middle to old age Controlling negative thoughts and staying calm Conclusions
**7**	**Fom Rabanal to Triacastela.** The Cognitive Distorters (Lucifer’s henchmen) attack Jacobo. Various allies teach Jacobo to replace his negative thoughts with more rational and positive alternatives using specific cognitive techniques: the direct approach, ‘‘the worst that could happen”, “survey” and “how does it help me to think like this”. Jacobo defeats the Cognitive Distorters.	**Contents:** Cognitive distortions (selective abstraction, mind reading, fortune-telling, “must” or “should” statements) Breaking negative thought patterns **Techniques/strategies:** - Feedback on task completion - Long-term memory: review of the previous module - Cognitive restructuring strategies (direct approach, the worst that can happen, survey, how does it help me to think like this) - Attention, working memory, long-term memory, ideomotor praxis, cognitive function (planning) - Social skills, assertive communication - Positive reinforcement when completing microgames	**Tasks between modules (app):** The previous ones plus the following: Implementing and recording cognitive restructuring strategies	**Cognitive processes** **Contents:** Cognitive processes Cognitive changes in middle to old age Cognitive impairment Relevance of cognitive training Conclusions
**8**	**From Triacastela to Santiago de Compostela.** In the final stage, Jacobo is taught how to cultivate and strengthen the tree of self-esteem. Lucifer appears, with whom Jacobo battles. All the characters that Jacobo has encountered throughout the journey reappear, each contributing their teachings to help him defeat Lucifer. Jacobo then makes an offering to Saint James, who grants him the Compostela (certificate of completion of the Way of Saint James) as recognition for completing the pilgrimage and acquiring healthy lifestyle habits. Jacobo sets out to begin a new life equipped with more optimism, self-knowledge, and practical strategies for well-being.	**Contents:** Strengthening self-esteem Review of learned content **Techniques/strategies:** - Feedback on task completion - Long-term memory: review of the previous module - Techniques to increase positive thoughts (distraction and priming) - Techniques to strengthen self-esteem (achievement tree: identifying personal qualities and achievements) - Attention, ideomotor praxis, language, working memory, planning, long-term memory - Social skills, assertive communication - Positive reinforcement when completing microgames - Review of learned content - Maintenance of progress and farewell		**Social relations** **Contents:** Social relations Social relationships in middle to old age Primary and secondary social relations The impact of social relationships on health Conclusions

**Table 2:** Sociodemographic characteristics of the sample.

Characteristics	Total *n* = 556		CBI-V *n* = 279		CG *n* = 277	
	*n*	%	*n*	%	*n*	%
**Sex**						
Male	143	25.7	78	28.0	65	23.5
Female	413	74.3	201	72.0	212	76.5
**Age**						
M (SD)	60.8 (8.0)		61.0 (7.8)		60.6 (8.1)	
Range	45–85		45–85		45–81	
**Marital status**						
Single	197	35.4	95	34.0	102	36.8
Partnered	359	64.6	184	66.0	175	63.2
**Education level**						
Primary	82	14.7	36	12.9	46	16.6
Secondary	149	26.8	79	28.3	70	25.3
University	325	58.5	164	58.8	161	58.1
**Main activity**						
Bachelor’s, certificate holders, technicians, artists	152	27.3	81	29.0	71	25.6
Skilled and unskilled workers	97	17.5	48	17.2	49	17.7
Homemakers, unemployed, retired	307	55.2	150	53.8	157	56.7
**Monthly family income**						
≤EUR 999	59	10.6	31	11.1	28	10.1
EUR 1000–EUR 1999	194	34.9	102	36.6	92	33.2
≥EUR 2000	303	54.5	146	52.3	157	56.7

*Notes*: CBI-V = cognitive–behavioral intervention via interactive, multimedia, online, and serious video game with complementary smartphone app; CG = control group.

**Table 3:** Intervention effects on pre-post comparisons per group (within-group effects).

CBI-V Group (*n* = 279)		Pre- Intervention *M (SD)*	Post- Intervention *M (SD)*	*t*	*p*	Cohen’s *d* (95% CI)
SF-36	General Health	66.7 (1.2)	70.5 (1.2)	−3.836	<0.001	−0.195 (−0.295, −0.095)
	Body Pain	73.0 (1.4)	73.8 (1.4)	−0.662	0.508	−0.037 (−0.149, 0.074)
	Physical Functioning	84.5 (1.0)	85.4 (1.0)	−1.169	0.243	−0.060 (−0.162, 0.041)
	Physical Role	80.5 (1.9)	81.5 (2.0)	−0.453	0.650	−0.029 (−0.156, 0.098)
	Vitality	65.2 (1.2)	66.8 (1.3)	−1.475	0.141	−0.076 (−0.177, 0.025)
	Social Functioning	88.5 (1.2)	86.9 (1.3)	1.204	0.229	0.076 (−0.048, 0.200)
	Emotional Role	86.0 (1.9)	83.8 (1.9)	1.110	0.267	0.071 (−0.054, 0.196)
	Mental Health	76.2 (1.0)	78.7 (1.1)	−2.469	0.014	−0.138 (−0.247, −0.028)
	Physical Summary Index	48.6 (0.5)	49.3 (0.5)	−1.735	0.083	−0.090 (−0.192, 0.012)
	Mental Summary Index	50.5 (0.6)	50.4 (0.6)	0.058	0.953	0.003 (−0.108, 0.114)
Lifestyle habits	SHI	11.9 (0.3)	10.3 (0.3)	5.959	<0.001	0.319 (0.214, 0.424)
	BPAAT	3.5 (0.1)	4.3 (0.2)	−5.423	<0.001	−0.322 (−0.439, −0.206)
	REAP-S	32.6 (0.2)	34.0 (0.2)	−6.122	<0.001	−0.419 (−0.553, −0.285)
**CG** **(*n* = 277)**		**Pre-** **Intervention** ***M (SD)***	**Post-** **Intervention** ***M (SD)***	***t***	***p***	**Cohen’s *d* (95% CI)**
SF-36	General Health	66.6 (1.2)	65.7 (1.2)	0.975	0.330	0.050 (−0.050, 0.149)
	Body Pain	72.2 (1.4)	70.1 (1.4)	1.592	0.112	0.092 (−0.021, 0.205)
	Physical Functioning	85.4 (1.0)	83.6 (1.0)	2.115	0.035	0.110 (0.008, 0.212)
	Physical Role	80.3 (1.9)	76.4 (2.0)	1.837	0.067	0.123 (−0.008, 0.254)
	Vitality	65.6 (1.3)	64.8 (1.3)	0.771	0.441	0.042 (−0.066, 0.151)
	Social Functioning	85.1 (1.2)	83.4 (1.3)	1.270	0.204	0.084 (−0.046, 0.215)
	Emotional Role	83.1 (1.9)	80.4 (2.0)	1.314	0.189	0.085 (−0.042, 0.213)
	Mental Health	74.4 (1.1)	74.8 (1.1)	−0.459	0.646	−0.027 (−0.140, 0.087)
	Physical Summary Index	49.0 (0.5)	47.9 (0.5)	2.478	0.013	0.132 (0.027, 0.236)
	Mental Summary Index	49.0 (0.6)	48.7 (0.7)	0.548	0.584	0.032 (−0.084, 0.149)
Lifestyle habits	SHI	12.3 (0.3)	12.0 (0.3)	1.030	0.304	0.319 (0.214, 0.424)
	BPAAT	3.8 (0.1)	3.9 (0.1)	−0.239	0.811	−0.014 (−0.129, −0.101)
	REAP-S	32.9 (0.2)	33.0 (0.2)	−0.672	0.502	−0.047 (−0.182, 0.089)

*Notes*: CBI-V = cognitive–behavioral intervention via interactive, multimedia, online, and serious video game with complementary smartphone app; CG = control group. BPAAT = Brief Physical Activity Assessment Tool; REAP-S = Rapid Eating Assessment for Participants-Short Version; SF-36 = The 36-item Short-Form Health Survey; SHI = Sleep Hygiene Index.

**Table 4:** Intervention effects on post-intervention comparisons between groups (between-group effects).

Comparison CBI-V vs. CG		*t*	*p*	Cohen’s *d* (95% CI)
SF-36	General Health	−2.760	<0.001	−0.248 (−0.424, −0.072)
	Body Pain	−1.898	0.058	−0.165 (−0.336, 0.006)
	Physical Functioning	−1.281	0.201	−0.114 (−0.289, 0.061)
	Physical Role	−1.783	0.075	−0.161 (−0.338, 0.016)
	Vitality	−1.129	0.259	−0.099 (−0.272, 0.073)
	Social Functioning	−1.945	0.052	−0.172 (−0.346, 0.002)
	Emotional Role	−1.229	0.219	−0.108 (−0.279, 0.064)
	Mental Health	−2.450	0.015	−0.218 (−0.392, −0.043)
	Physical Summary Index	−1.928	0.054	−0.171 (−0.345, 0.003)
	Mental Summary Index	−1.877	0.061	−0.165 (−0.338, 0.008)
Lifestyle habits	SHI	3.779	<0.001	0.334 (0.160, 0.507)
	BPAAT	−2.261	0.024	−0.188 (−0.351, −0.025)
	REAP-S	−3.205	0.001	−0.291 (−0.469, −0.113)

*Notes*: CBI-V = cognitive–behavioral intervention via interactive, multimedia, online video game with complementary smartphone app; CG = control group. BPAAT = Brief Physical Activity Assessment Tool; REAP-S = Rapid Eating Assessment for Participants-Short Version; SF-36 = The 36-item Short-Form Health Survey; SHI = Sleep Hygiene Index.

**Table 5:** Percentage of participants who showed clinical significance.

Variables		CBI-V *n* = 279		CG *n* = 277		χ^2^	*p*
		*n*	%	*n*	%		
SF-36	**General Health**						
	No clinically significant change	89	31.9	113	40.8	4.75	0.029
	Clinically significant change	190	68.1	164	59.2		
	**Body Pain**						
	No clinically significant change	157	56.3	167	60.3	0.92	0.337
	Clinically significant change	122	43.7	110	39.7		
	**Physical Functioning**						
	No clinically significant change	50	17.9	71	25.6	4.85	0.028
	Clinically significant change	229	82.1	206	74.4		
	**Physical Role**						
	No clinically significant change	75	26.9	82	29.6	0.51	0.476
	Clinically significant change	204	73.1	195	70.4		
	**Vitality**						
	No clinically significant change	89	31.9	108	39.0	3.05	0.081
	Clinically significant change	190	52.9	169	61.0		
	**Social Functioning**						
	No clinically significant change	123	44.1	152	54.9	6.47	0.011
	Clinically significant change	156	55.9	125	45.1		
	**Emotional Role**						
	No clinically significant change	80	28.7	109	39.4	7.06	0.008
	Clinically significant change	199	71.3	168	60.6		
	**Mental Health**						
	No clinically significant change	67	24.0	90	32.5	4.93	0.026
	Clinically significant change	212	76.0	187	67.5		
	**Physical Summary Index**						
	No clinically significant change	59	21.1	85	30.7	6.59	0.010
	Clinically significant change	220	78.9	192	69.3		
	**Mental Summary Index**						
	No clinically significant change	68	24.4	84	30.3	2.48	0.115
	Clinically significant change	211	75.6	193	69.7		
Lifestyle habits	**Sleep hygiene**						
	No clinically significant change	2	0.7	10	3.6	5.39	0.020
	Clinically significant change	275	99.3	269	96.4		
	**Physical activity**						
	No clinically significant change	85	30.5	104	37.5	3.11	0.078
	Clinically significant change	194	69.5	173	62.5		
	**Eating habits**						
	No clinically significant change	92	33.0	118	42.6	5.48	0.019
	Clinically significant change	187	67.0	159	57.4		

*Notes*: CBI-V = cognitive–behavioral intervention via interactive, multimedia, online serious video game with complementary smartphone app; CG = control group.

## Data Availability

The data presented in this study are available on request from the corresponding author. The data are not publicly available because they contain sensitive patient information.

## Abbreviations

The following abbreviations are used in this manuscript:

BPAAT	Brief Physical Activity Assessment Tool for Primary Care Consultations
CBI-V	Cognitive-behavioral intervention via an interactive multimedia online video game with a complementary smartphone app
CG	Control group
CSQ-8	Client Satisfaction Questionnaire
GAMAPEA	*Gamificación aplicada a la promoción del envejecimiento activo* [Gamification applied to the promotion of active aging]
LMM	Linear Mixed Models
MINI	Mini International Neuropsychiatric Interview
MMSE	Mini-Mental State Examination
RCT	Randomized Controlled Trial
REAP-S	Rapid Eating Assessment for Participants-Short Version
SF-36	Short-Form Health Survey
SHI	Sleep Hygiene Index
WHO	World Health Organization
